# Techniques for Capturing Clinical Photographs in Orthodontics

**DOI:** 10.7759/cureus.73629

**Published:** 2024-11-13

**Authors:** Al Imran Shahrul, Nabilla Mohd Shukor, Noraina Hafizan Norman

**Affiliations:** 1 Department of Family Oral Health, Universiti Kebangsaan Malaysia, Kuala Lumpur, MYS; 2 Department of Orthodontics, Management and Science University (MSU) Medical Centre, Shah Alam, MYS; 3 Department of Orthodontics, Universiti Teknologi MARA, Shah Alam, MYS

**Keywords:** clinical records, digital imaging, digital photograph, orthodontic documentation, photo documentation

## Abstract

High-quality clinical photography is essential in orthodontics, playing a crucial role in diagnosis, treatment planning, patient education, and professional communication. However, capturing consistently clear and detailed orthodontic photographs can be challenging, particularly without knowledge of standardized techniques and equipment. This article provides a comprehensive guide on the key elements of effective orthodontic photography, including camera settings, positioning, and lighting. Illustrating each step in the process aims to equip orthodontists with the skills needed to achieve high-quality, reproducible clinical photos that enhance the quality of patient care and support clinical documentation.

## Introduction and background

Orthodontic clinical photos are an essential part of daily orthodontic practice [1,2]. Their benefits [3] include serving as a baseline record, assisting in communicating with general dental practitioners and patients, helping in treatment planning, assisting orthodontists in teaching, monitoring treatment progress, and medico-legal and research purposes. The difference between orthodontic clinical photos and other forms of photography is their purpose, which is to record true-to-life patient features rather than masking these features to make the photo more pleasing. The key goal of orthodontic clinical photos is taking sequential photos as consistently as possible [4-6]. This may sound relatively simple but is quite difficult to achieve in practice.

**Figure 1:**
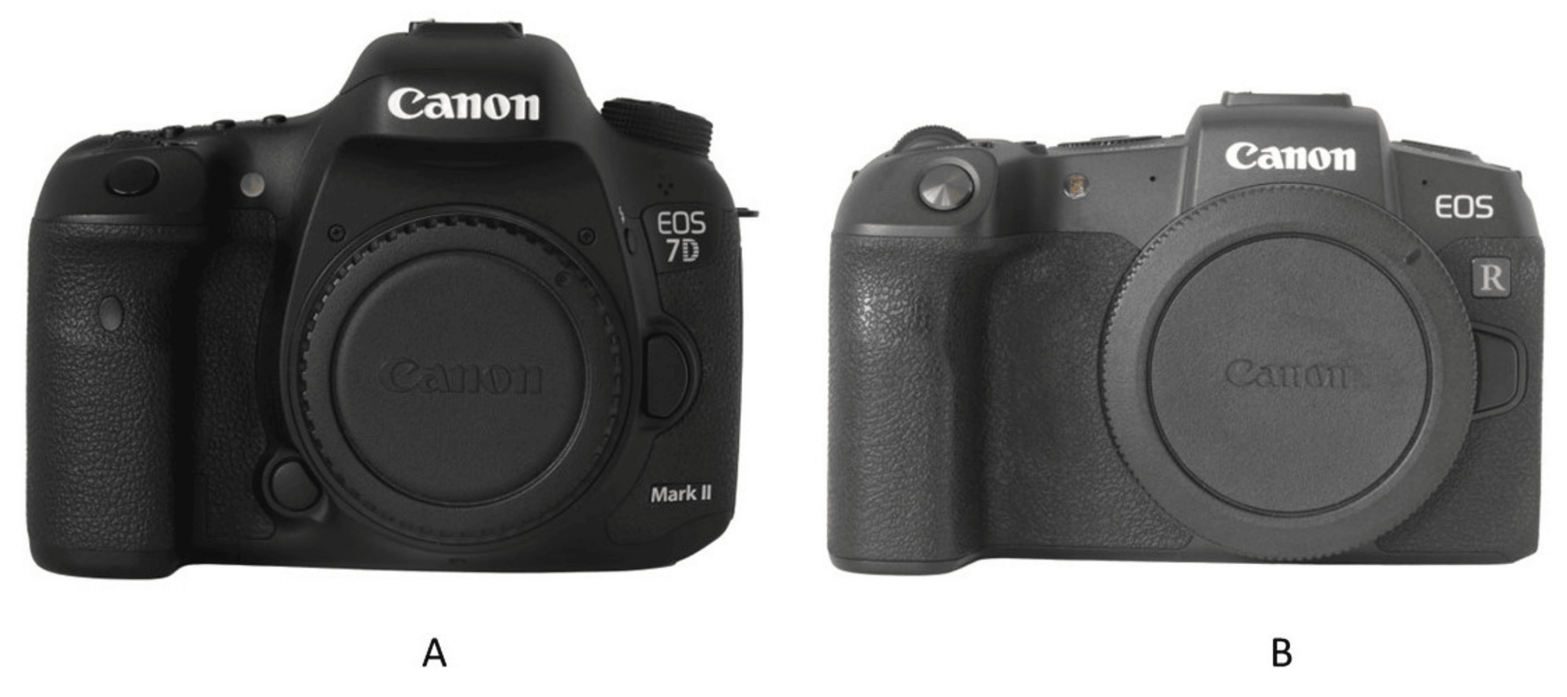
(A) Digital single-lens reflex and (B) mirrorless cameras.

**Figure 2:**
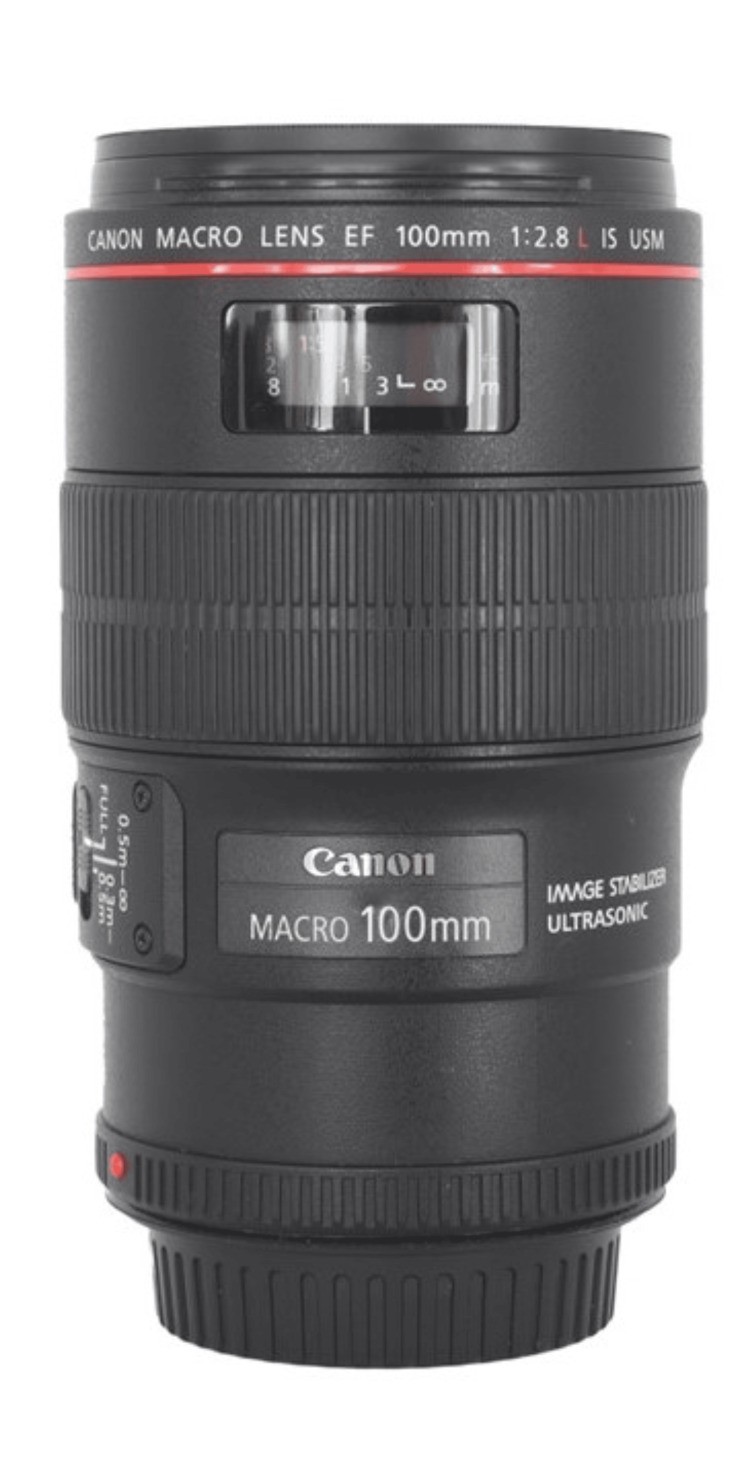
A macro lens with 100mm focal length.

A complete orthodontic record consists of extraoral and intraoral photos [7,8]. Obtaining consistent high-quality extraoral and intraoral orthodontic clinical photos requires the proper equipment, such as a camera, lens, lighting, retractor, and mirror [5,9]. The camera should be a digital single-lens reflex (DSLR) or mirrorless camera with an interchangeable lens (Figure 1) [4,10-13]. Any modern-day digital camera that was released in the past five years should be more than sufficient to take quality orthodontic photos. A macro lens with 100mm focal length is recommended (Figure 2) [9]. A macro lens allows focus on a close subject, and a 100mm focal length produces less lens distortion. The lighting, retractor, and mirror will be discussed in more detail in the next sections.

In this era of digital cameras, the orthodontist has the flexibility to manipulate and crop images [14]. However, it is best to get the photo correct in the camera. Cropping the image can affect the photo magnification, thereby affecting the consistency of the photos. The camera equipment is just a tool like any other orthodontic instrument. Orthodontists can have the most advanced tools at their disposal, but without proper technique, they would not obtain a high-quality orthodontic photo. In this article, the author attempts to provide the correct technique required for orthodontists to take high-quality orthodontic clinical photos.

## Review

Extraoral photos

General Guide

**Figure 3:**
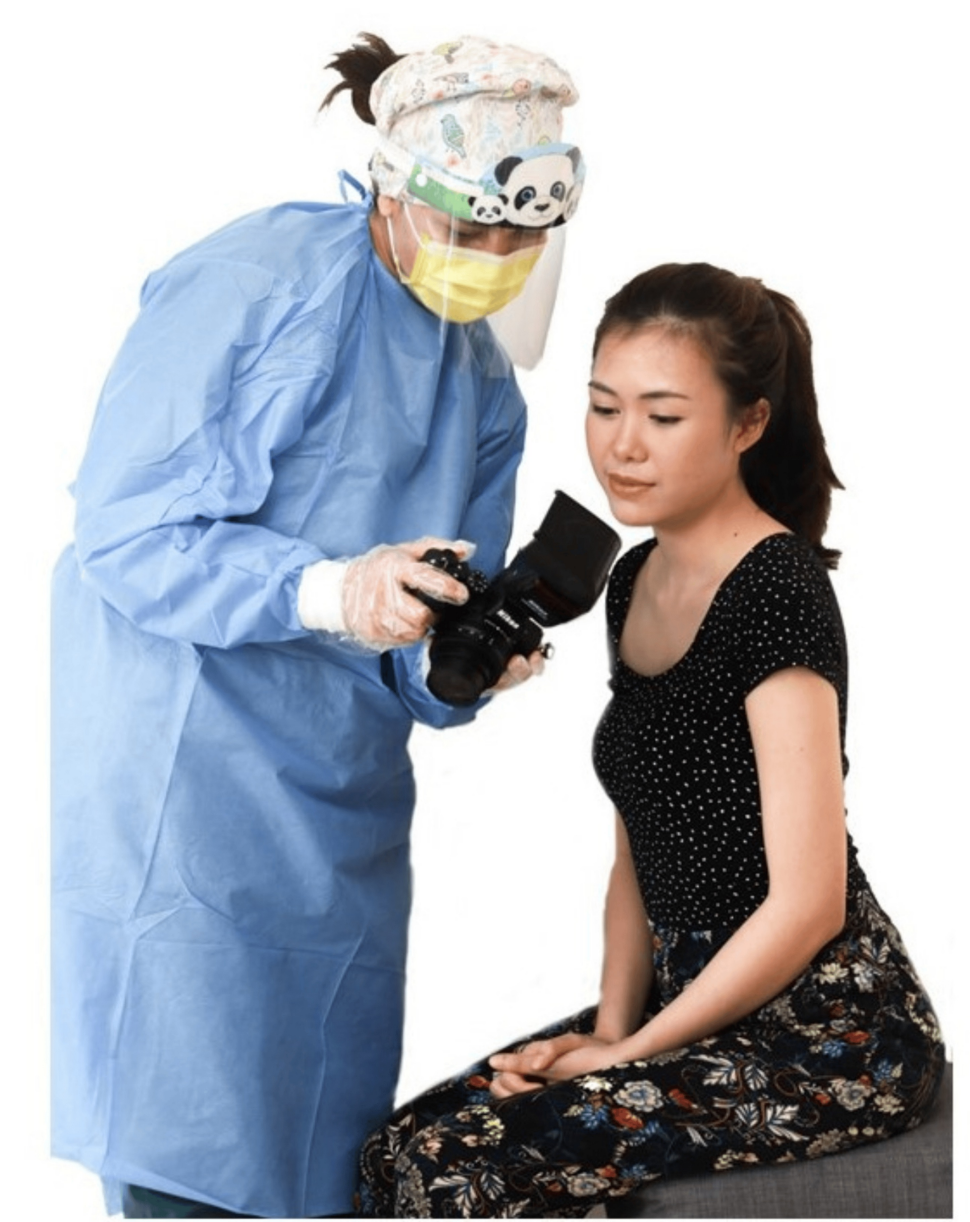
An orthodontist explaining the extraoral photo procedure to a patient. Consent was obtained from the volunteers for the use of the images.

Extraoral photos are typically taken on the patient’s first visit to the orthodontist. It is advisable to start record taking with extraoral photos [5], as it is the most familiar procedure and causes the least discomfort. The patient typically will experience more discomfort during impression taking for a study model, intraoral photos, and radiographs. The orthodontist will typically interact with the patient while taking extraoral photos to build rapport. It is best to show the patient an example of the type of photo to be taken and to explain the steps involved and what is expected from them (Figure 3). The patient is also typically excited during this time; hence, there is a greater possibility of getting a natural smile.

Attire

**Figure 4:**
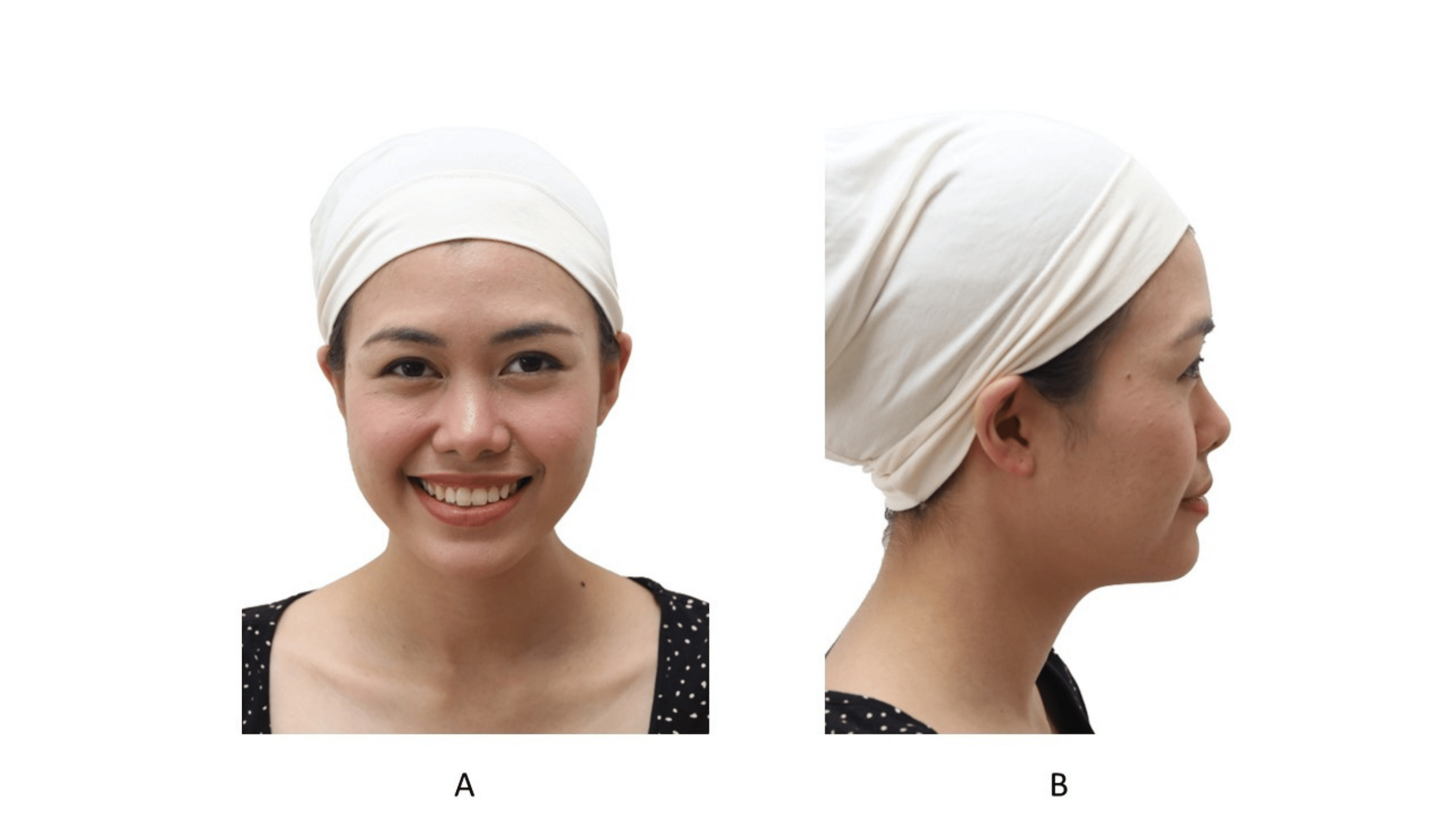
A patient with a head scarf allowing the jaw and ears to show. A: Frontal view; B: Profile view Consent was obtained from the volunteers for the use of the images.

**Figure 5:**
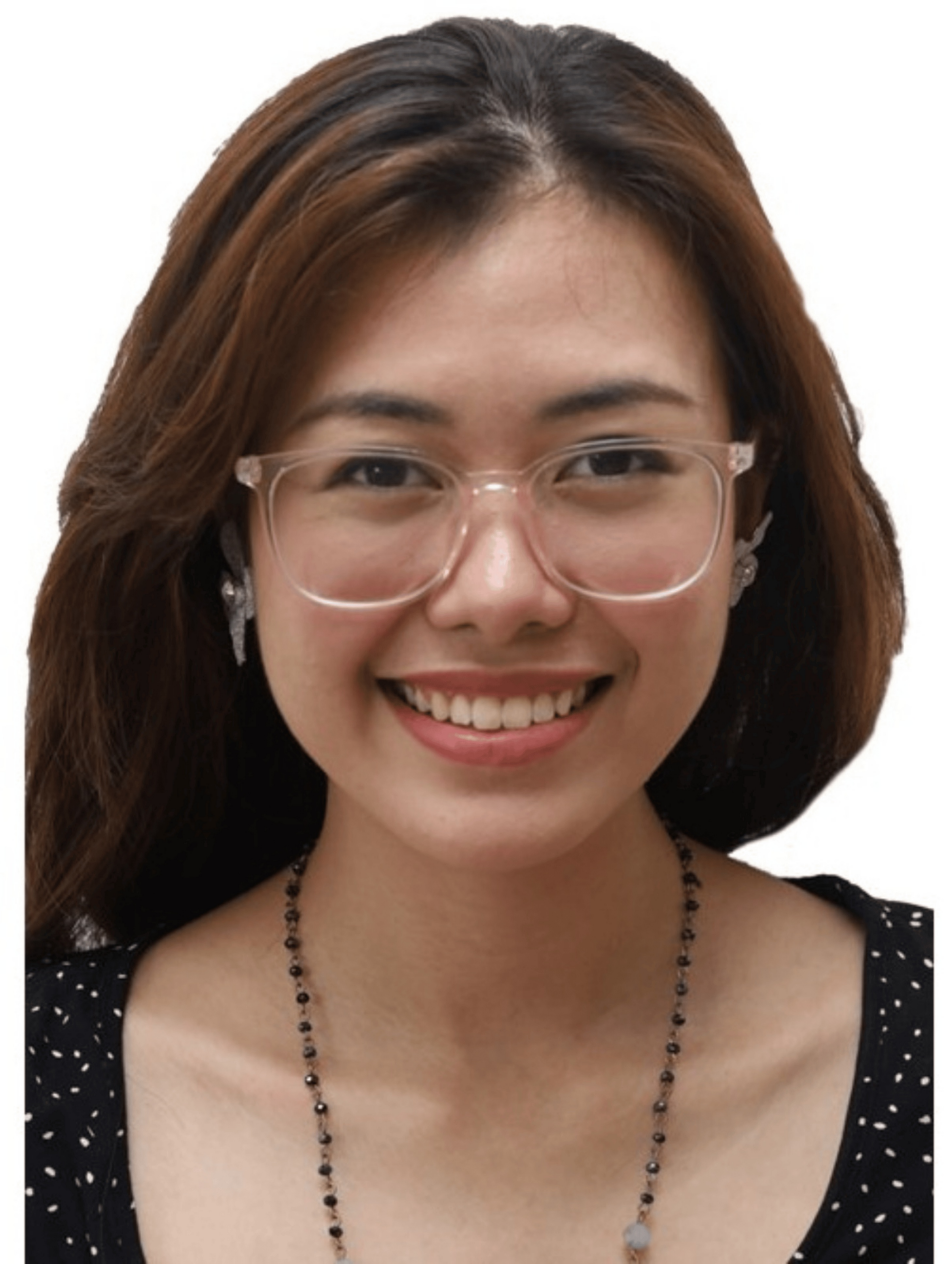
Unacceptable attire for an extraoral photo. Consent was obtained from the volunteers for the use of the images.

Any clothing that obscures the patient’s facial features should be removed [5,15]. Head scarves should be removed if it is acceptable. If a patient cannot remove their head scarf, the author provides the patient with a head scarf that exposes their jaw and ears (Figure 4). Patients should tie back long hair with a neutral-colored headband or hair scrunchie so as to not obscure the face and ears. Glasses, necklaces, piercings, and heavily applied makeup should be removed (Figure 5).

Background and Lighting

**Figure 6:**
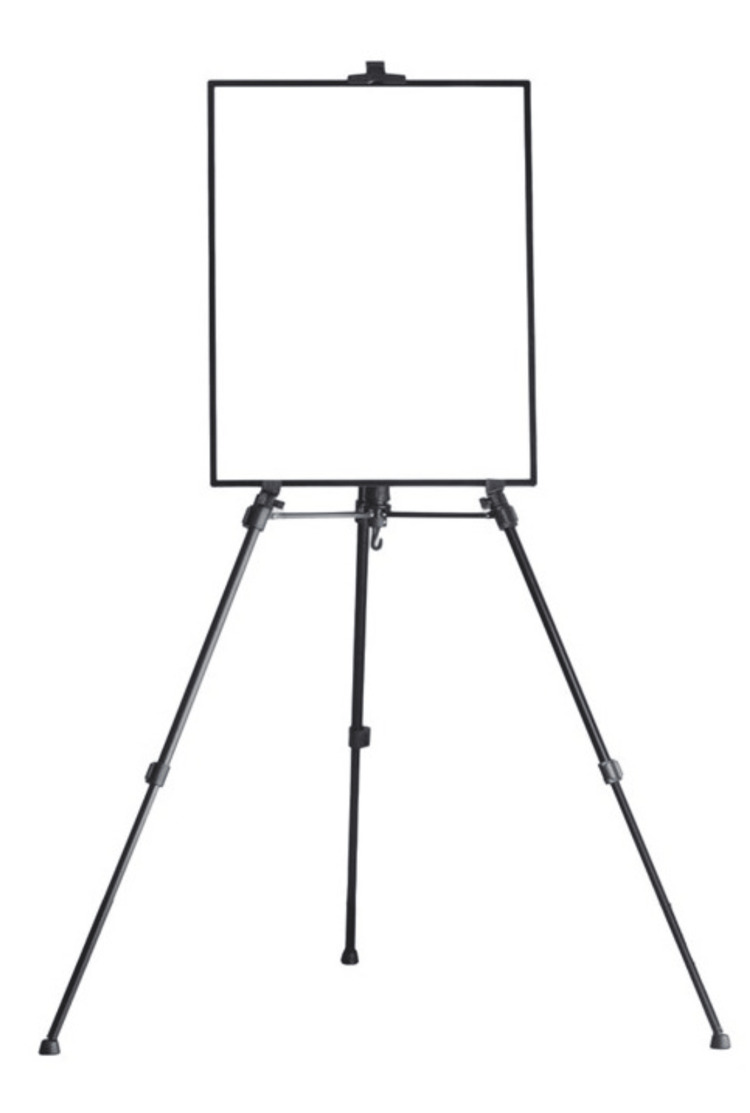
An LED light box can be used as a background.

**Figure 7:**
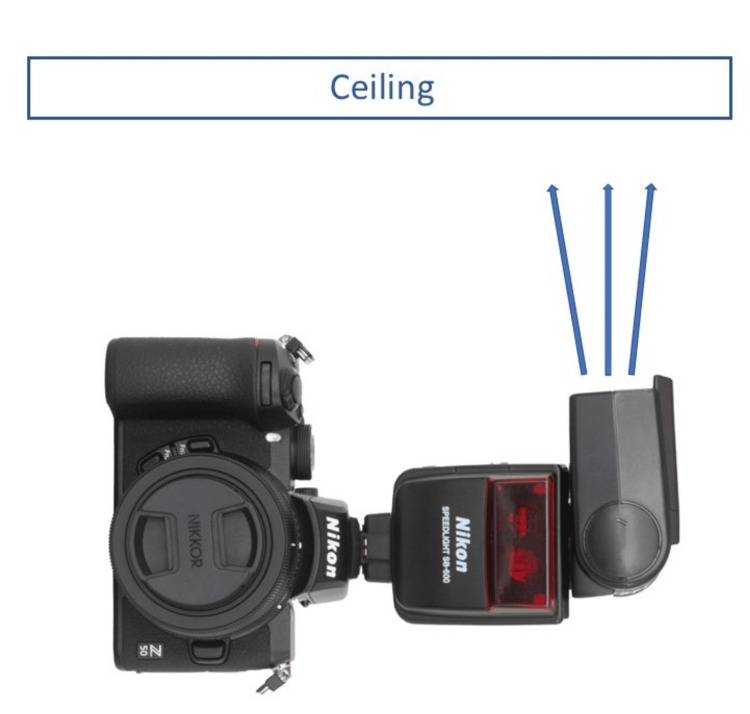
A camera in portrait orientation with the flash directed toward the ceiling.

**Figure 8:**
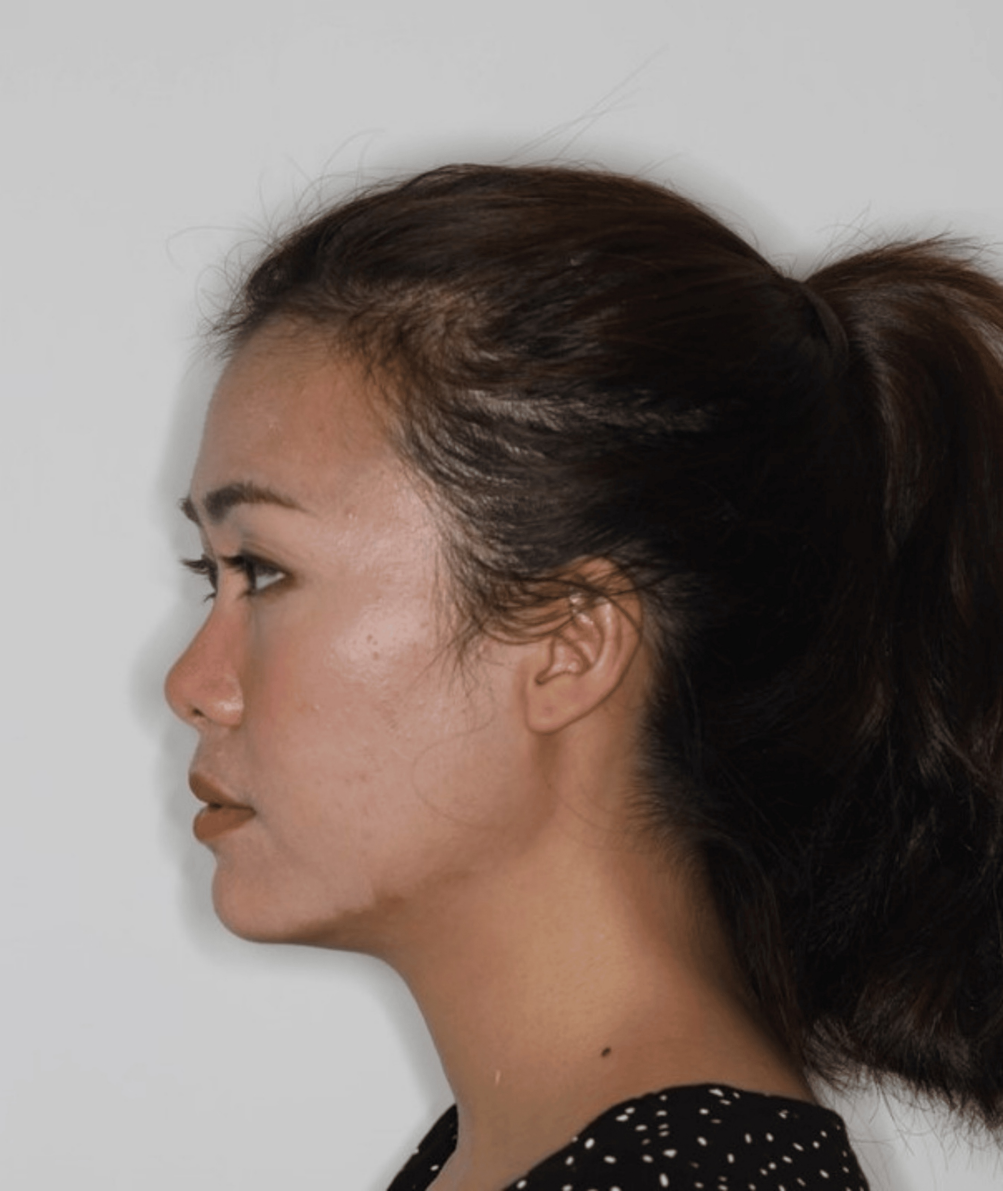
Photograph of a patient taken using a ring flash. Consent was obtained from the volunteers for the use of the images.

The background and the lighting setup under which the photos are taken must remain constant, as modification can affect the pre- and post-operative photos. A plain white or black background is advisable. However, a white background may produce shadows. There are two methods to overcome this. First, a light box can be used as the background (Figure 6). Second, an external flash with a rotating head directed to the ceiling can be used (Figure 7) [16]. Using the camera’s built-in flash or a ring flash is not advisable [16,17] because a flash pointing directly at the patient provides harsh lighting that produces shadows and a "red-eye" effect (Figure 8). To achieve the best result, use both a light box as a background and an external flash.

Positioning

**Figure 9:**
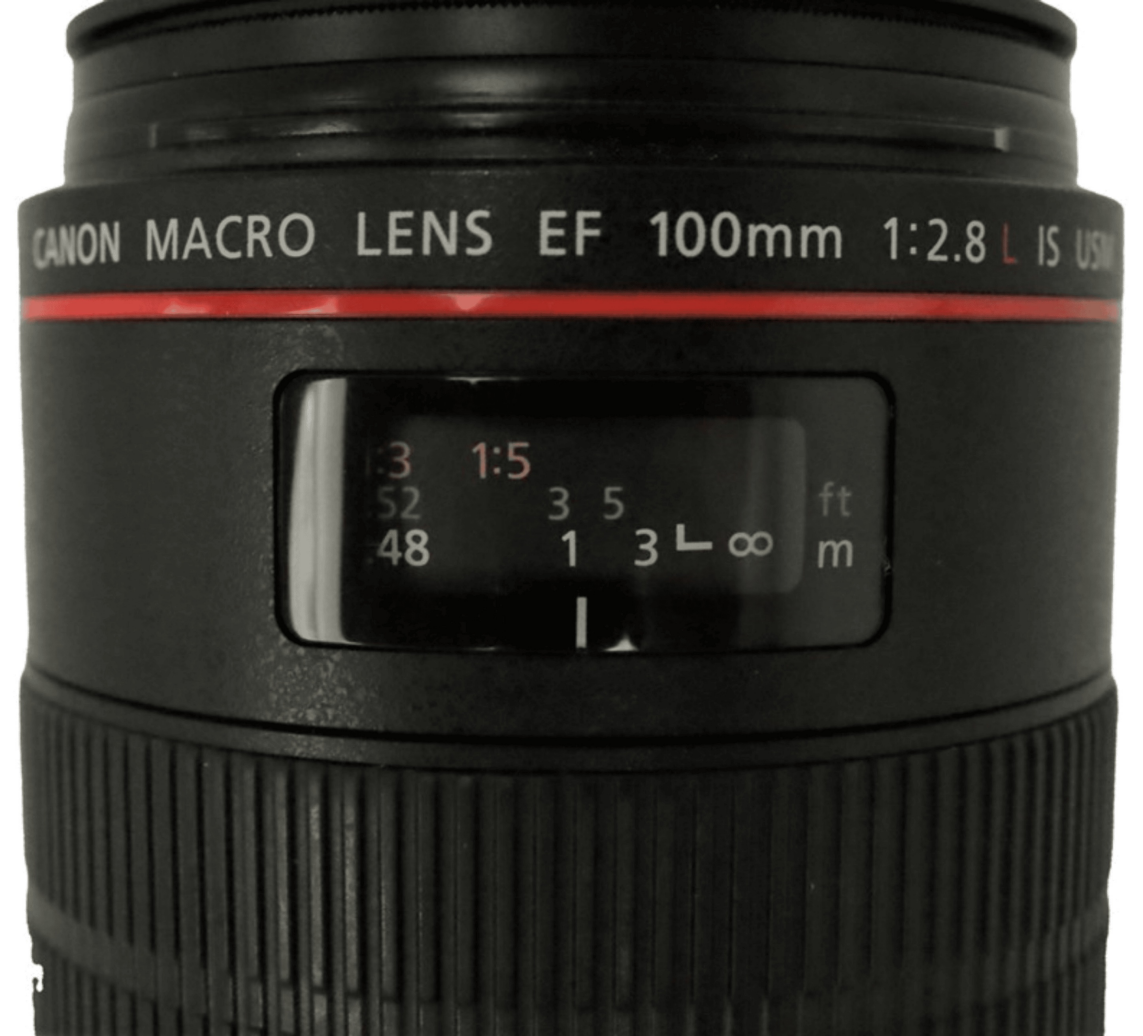
The focus distance window in a macro lens.

To produce consistent photos, all photos should be taken from the same distance [6]. To achieve this, the lens should be the same focal length and focus distance (Figure 9). The orthodontist also should use the same sensor size camera for each photo. A full-frame sensor-size camera will yield a different focal length than an Advanced Photo System type-C (APS-C) sensor-size camera. The focus distance can be set using the manual focus function, and then the clinician can move forward and backward until the image comes into focus. However, using manual focus with children can be challenging, as they often move and may not stay in the same position while the orthodontist adjusts for focus. In these situations, it is recommended that the orthodontist use the autofocus feature. To maintain a consistent distance for each patient when using autofocus, the orthodontist placed tape on the clinic floor at a predetermined distance, and the focus point should be positioned below the eyelid.

**Figure 10:**
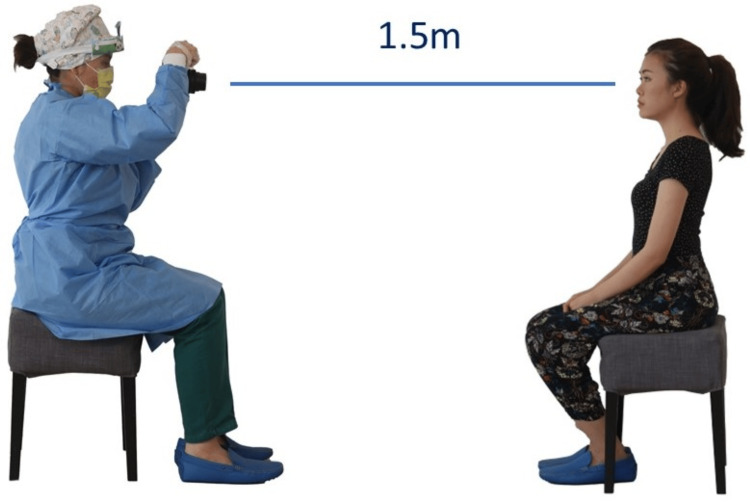
The position of the orthodontist and the patient during extraoral photos. Consent was obtained from the volunteers for the use of the images.

The position of the patient when the photo is taken must be kept as consistent as possible [6]. The patient should be standing during extraoral photos, as in the author’s experience patients tend to slump when seated. However, issues may arise due to a difference in height between the orthodontist and the patient. If this occurs, the author takes the photo from a seated position (Figure 10). The patient's chair should be stable. Swivel chairs should be avoided because children tend to turn the chair while having their photo taken.

Once seated, the patient should be in the natural head position [18]. The natural head position is the position in which the patient naturally carries their head. To attain this position, the patient is instructed to sit up straight and to look straight at a distant point at eye level. The patient’s Frankfort plane should be parallel to the floor [19]. Once the patient is in the correct position, the camera lens also should be parallel to the floor.

Camera Setting

The camera aperture should be set to f/8 [20,21] with a shutter speed of 1/200s and ISO of 100. The camera should be positioned in the portrait orientation. If an external flash is to be used it should be set to evaluative through-the-lens (ETTL) mode. If a ring flash is used, the flash should be set to ¼ power in a full-frame sensor camera or ½ power in an APS-C size sensor camera. The ring flash also should be set to produce a flash only on one side to reduce the amount of shadow produced.

Frontal Photo

**Figure 11:**
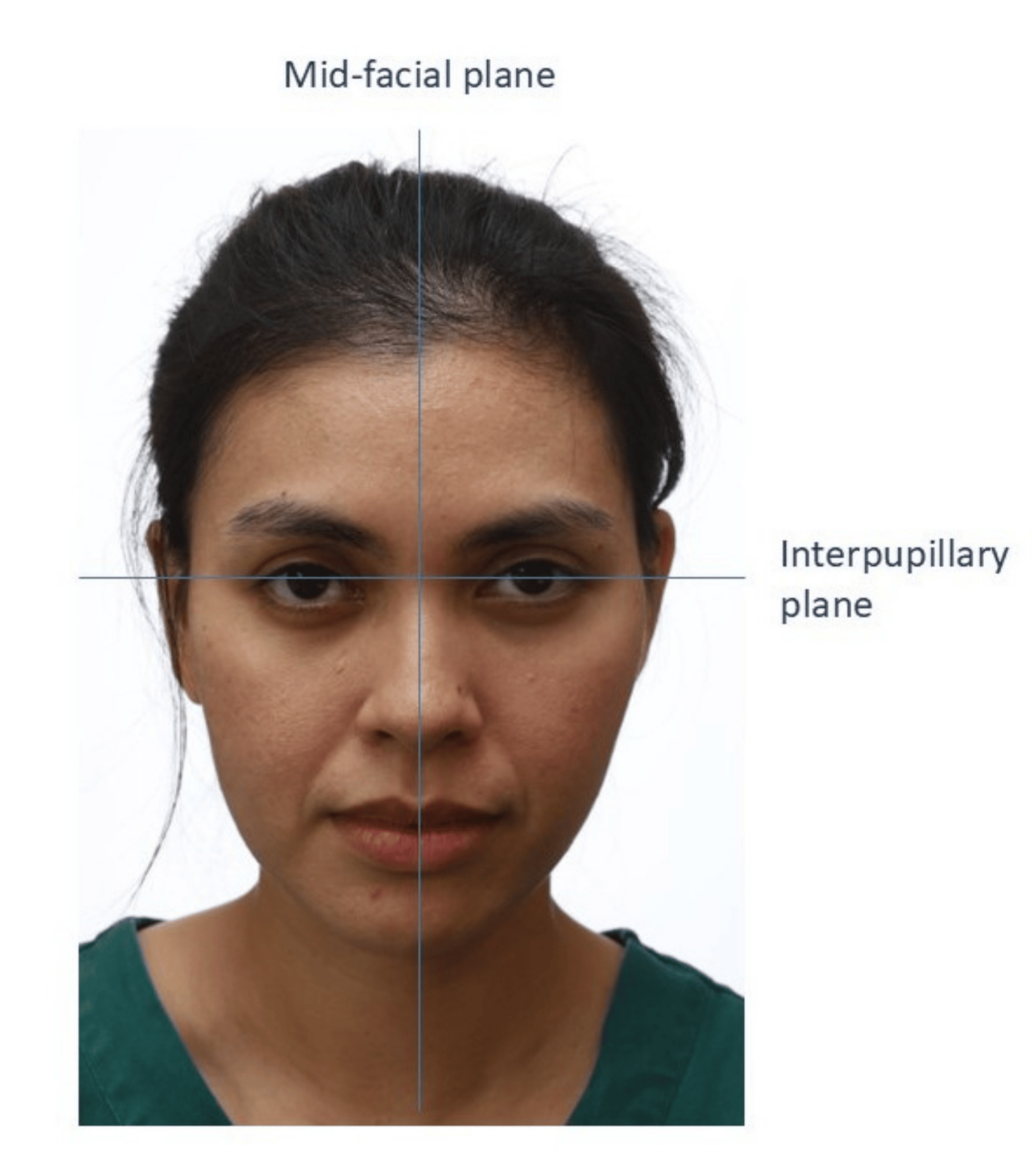
A patient in the frontal view. Consent was obtained from the volunteers for the use of the images.

Frontal photos typically consist of two types: at rest and smiling. The patient’s mid-facial plane should be at the center of the frame (Figure 11). The interpupillary plane should be parallel to the floor unless there is a facial asymmetry. For the at-rest photo, the patient should be lightly in occlusion. The clinician should detect if the patient is habitually trying to close their lips. Any lip incompetency or lip trap should be recorded. If there is asymmetry, an additional photo should be taken. The patient is instructed to bite on the occlusal plane. This will expose any canting of the occlusal plane in relation to the interpupillary plane.

**Figure 12:**
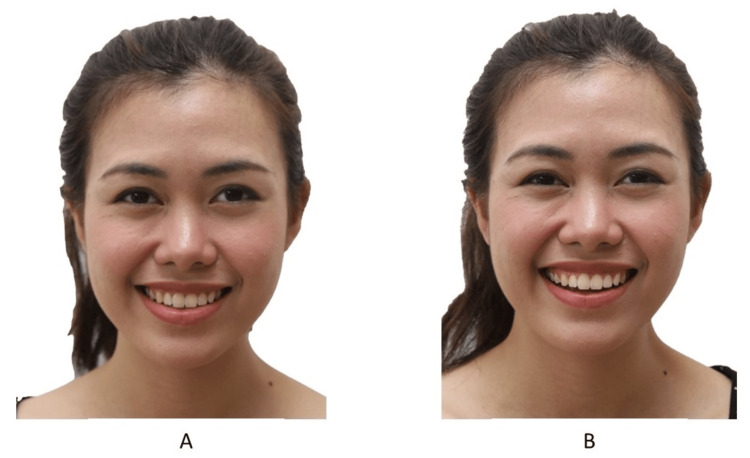
(A) The posed smile and (B) the spontaneous smile. Consent was obtained from the volunteers for the use of the images.

Two types of smiling photos should be taken: spontaneous and posed (Figure 12) [22,23]. The authors prefer to take a posed smile photo because it is reproducible and the smile that the patient sees when looking at a mirror or when taking photos. The spontaneous smile is involuntary and induced by laughter. The spontaneous smile also is more animated with an increase in gingival show. Getting a posed smile from a patient with malocclusion may be challenging. In the author’s experience, the patient tends to close their lips while smiling to prevent people from seeing their teeth. Good communication is essential during this procedure to help the patient feel comfortable smiling.

Profile Photo

**Figure 13:**
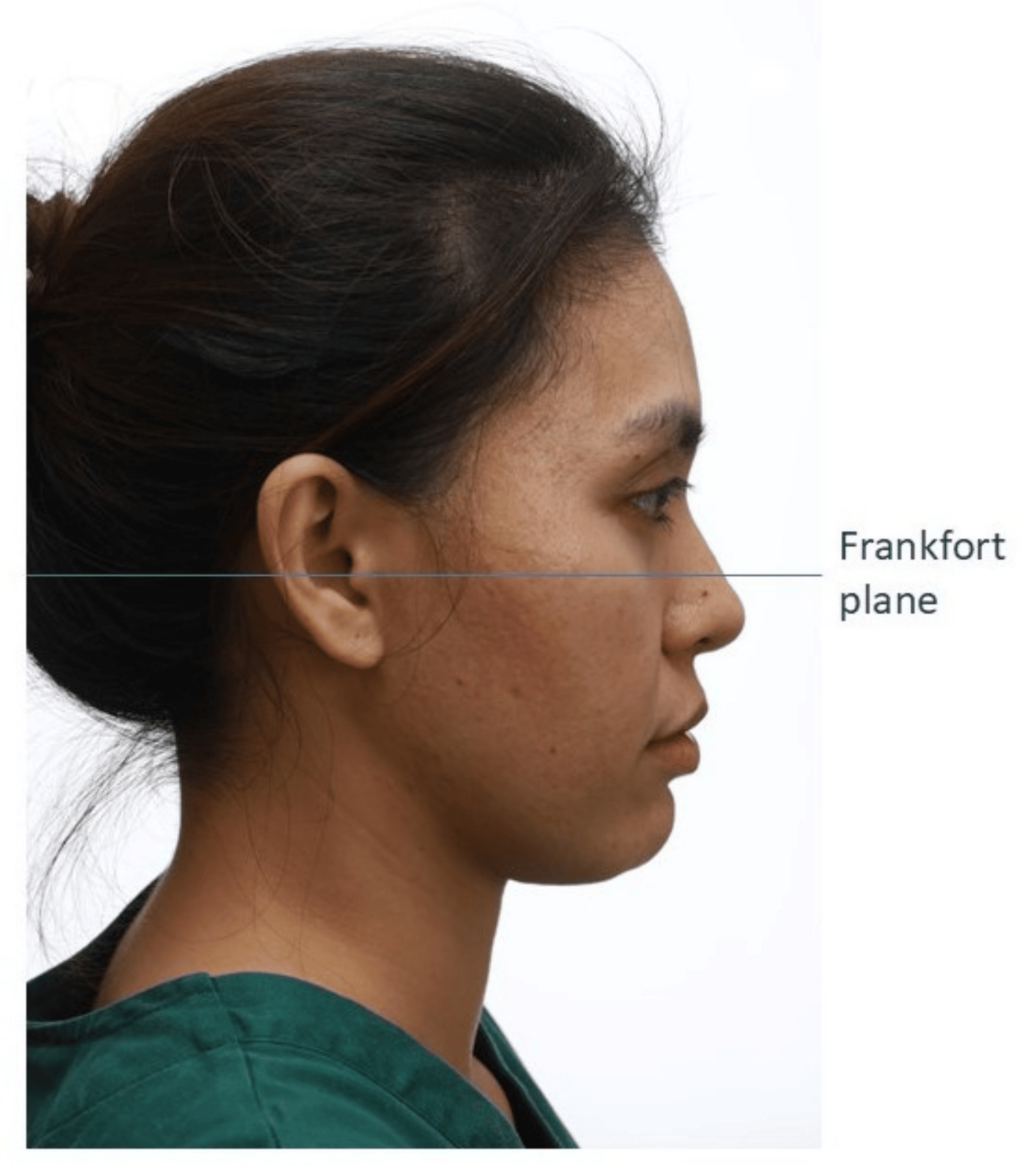
A patient in the profile view. Consent was obtained from the volunteers for the use of the images.

For the profile photo, the patient should be in the natural head position (Figure 13) [18]. Any tilting of the head will alter the patient’s sagittal appearance [6]. The patient’s occipital region should be included in the frame. If the patient has facial asymmetry, photos of both sides of the face should be taken. The clinician should make sure the patient does not habitually posture their mandible forward, especially patients with a large overjet. If there is any anterior crossbite with mandibular displacement, two photos in centric relation and centric occlusion must be taken to record the amount of anterior displacement.

Three-Quarter View

**Figure 14:**
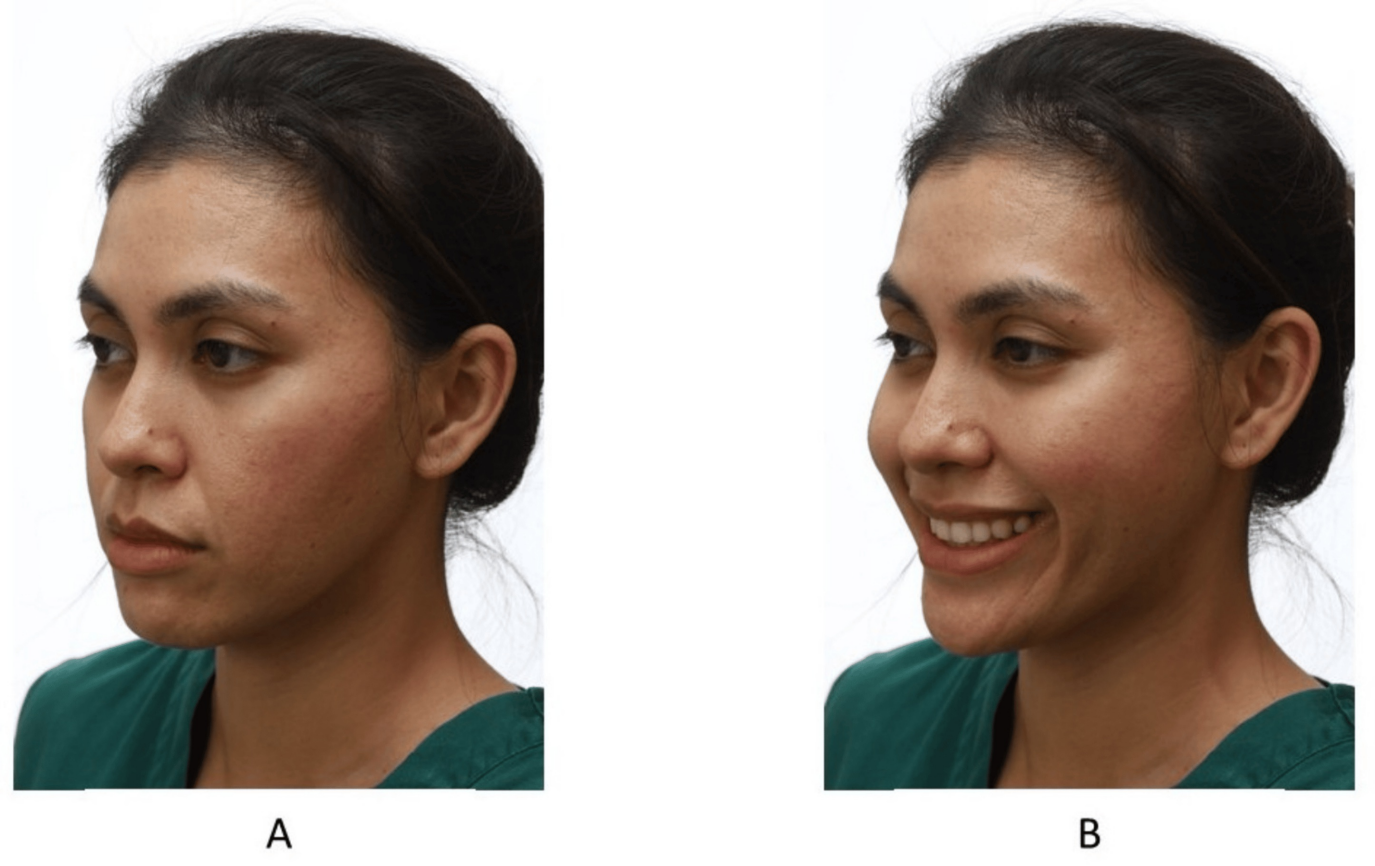
A patient in the three-quarter view, (A) relaxed and (B) smiling. Consent was obtained from the volunteers for the use of the images.

The three-quarter view is taken occasionally from one side only. There are two ways of positioning the patient (Figure 14). In one, the entire body is positioned at a turn of 45 degrees. In an alternative way, the patient turns the head 45 degrees while keeping the body perpendicular to the camera. The author prefers the prior method because the patient’s head is in a more relaxed position. Once the patient has been properly positioned, two photos are typically taken. In one, the patient is in a relaxed position, while in the other the patient is smiling [22].

Intraoral photos

General Guide

Prior to taking orthodontic photos, make sure the patient’s teeth are clean from any food remnants [24]. For treatment progress photos, the clinician is advised to remove any debris stuck within the appliance, especially in the molar buccal accessory tube or in the doors of self-ligating braces. Pooling saliva should be removed using low-volume suction [25]. In addition, it is advisable to take photos for treatment progress records after the reactivation or placement of new mechanics.

For the first-time patient, it is best to show the patient the retractor and mirror that will be used prior to taking intraoral photos, especially for children. Children should be motivated and congratulated throughout the procedure. The author finds the tell-show-do method very helpful.

Retractor

**Figure 15:**
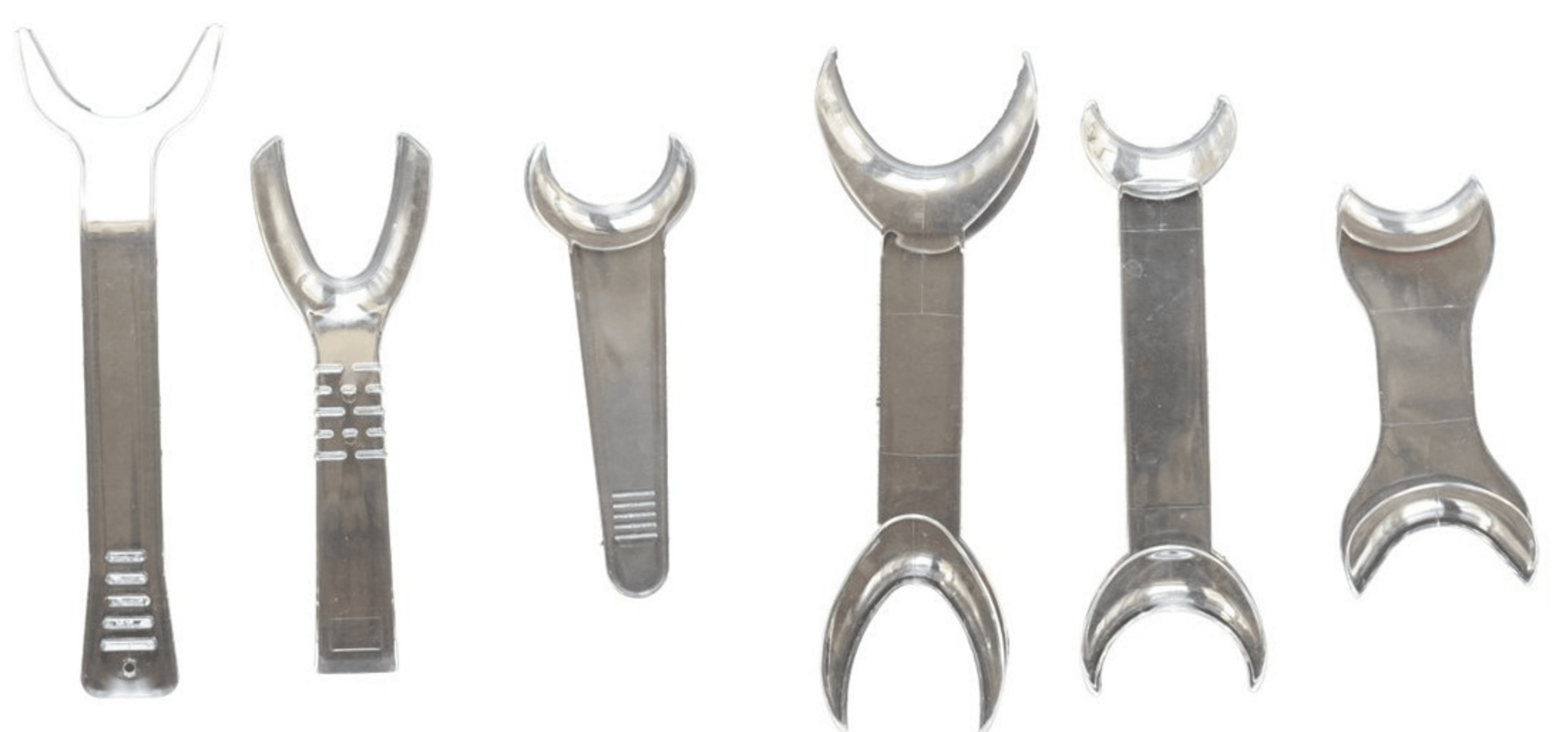
Various cheek retractors.

No one retractor fits all patient types [5,9,24,26]. The clinician requires multiple retractor sizes and shapes for intraoral photos (Figure 15). Prior to the placement of the retractor, the author applies Vaseline around the patient’s lips [27] to aid the placement, or asks the patient to lick their own lips to wet them. A clear retractor is preferred because it is less distracting than a black or white retractor. Pulling the retractor can cause discomfort to the patient; hence, the author will typically achieve focus and framing before retracting. If the patient has difficulty biting, the clinician probably used a retractor that was too large.

Mirror

**Figure 16:**
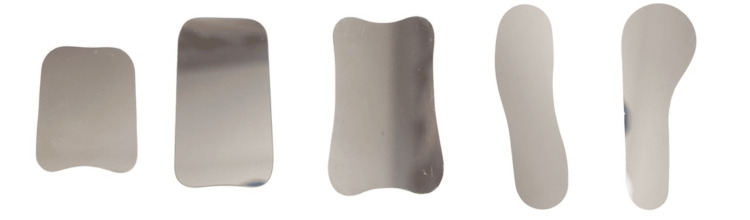
Various mirrors.

Various sizes and types of mirrors are available. The clinician should have sizes to fit each patient (Figure 16). A mirror with a handle is preferred [9,26]. Over time, the mirror may get scratched. If these scratches interfere with the photo quality, a replacement is necessary. The retractor should be inserted prior to the mirror. Insertion should be like an impression tray, from the side and then rotated inside the mouth. To avoid fogging, the mirror can be warmed prior to insertion. It is recommended that the orthodontist use the three-way syringe to blow air onto the intraoral mirror, preventing fogging and clearing any saliva immediately before taking a photo [27]. Instructing the patient to hold their breath immediately before taking a photo can aid in preventing fogging.

Lighting

**Figure 17:**
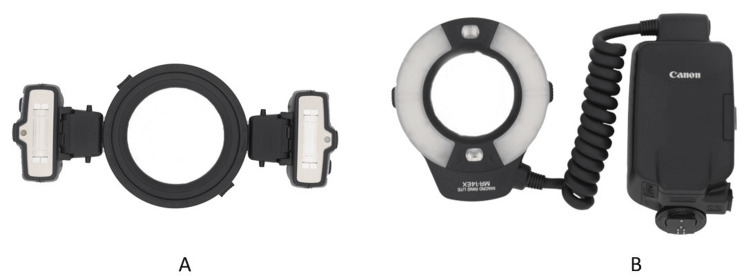
(A) A twin flash and (B) a ring flash.

Intraoral photos require additional lighting [24]. The author recommends a ring or twin flash for this purpose (Figure 17) [16]. The ring is preferred due to its simplicity. The camera’s built-in flash is not suitable because it is positioned farther from the lens and provides less output of light. The dental chair light should not be used to illuminate the teeth.

Positioning

All intraoral photos are taken on the dental chair. The clinician should stand while taking the photos. The patient should be positioned 45 degrees from the floor for anterior and buccal photos. For occlusal photos, the patient should be in a supine position [24]. The dental assistant should be positioned at 2 o’clock to aid visibility when retracting.

Camera Setting

**Figure 18:**
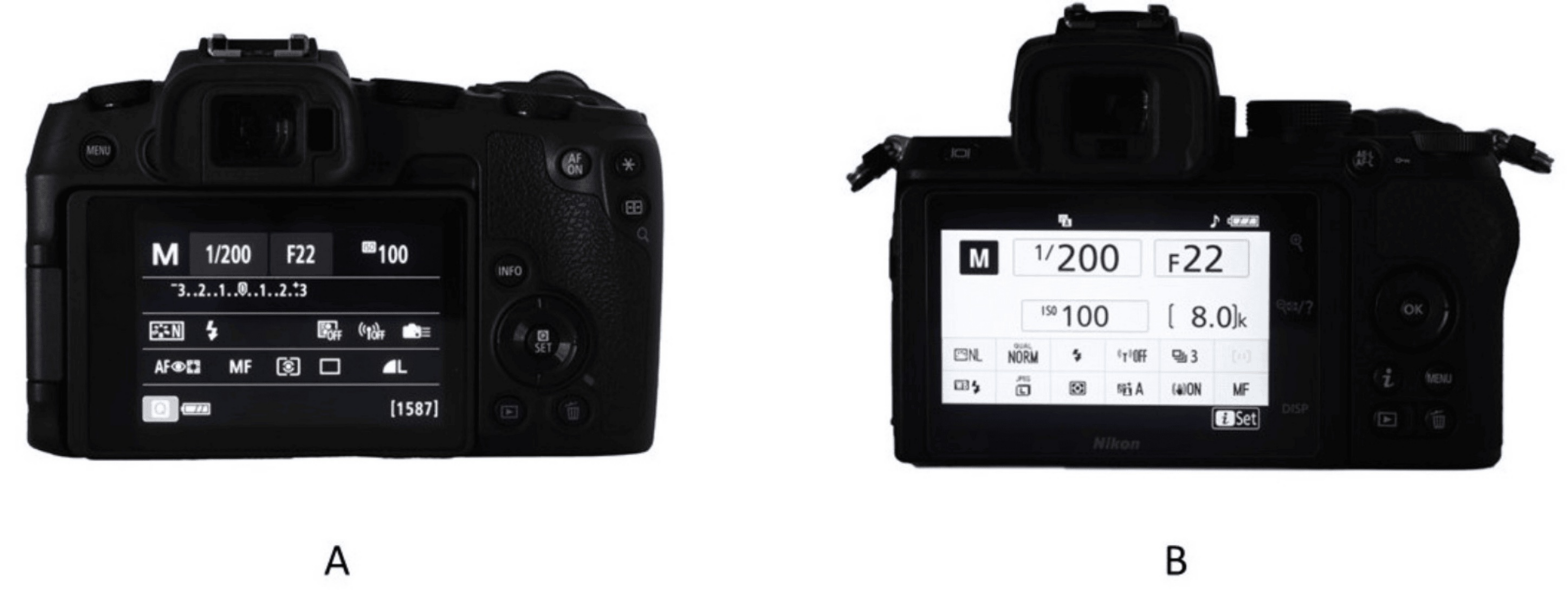
The recommended settings for intraoral photos. A: Full-frame camera; B: APS-C camera APS-C: Advanced Photo System type-C

The camera aperture should be set to f/22 [20] with a shutter speed of 1/200s and ISO of 100. The camera should be positioned in the landscape orientation (Figure 18). The ring flash should be set to ¼ power in a full-frame sensor camera or ½ power in an APS-C size sensor camera.

Anterior Photo

**Figure 19:**
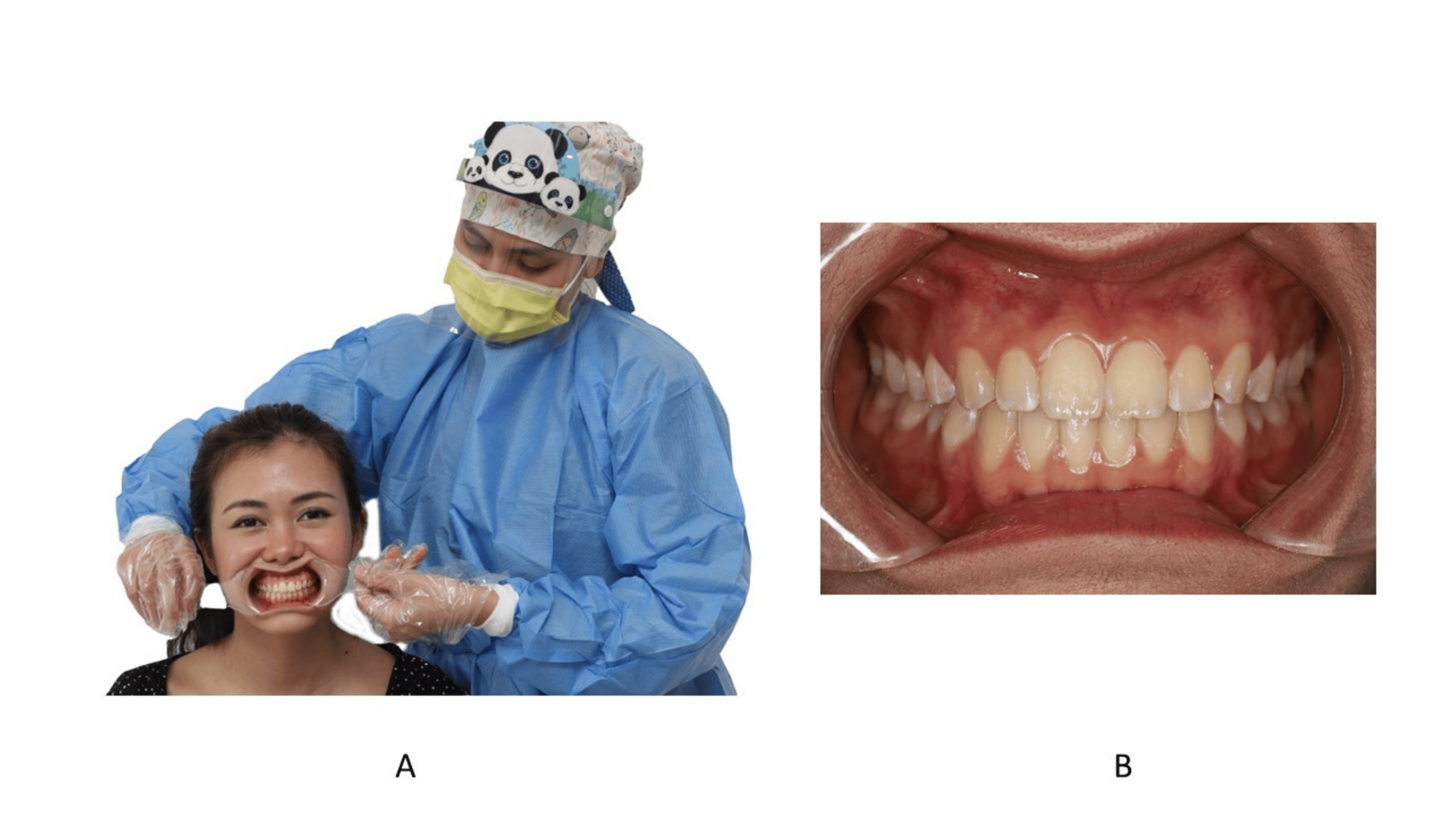
The position of the retractor during anterior view. A: Front view; B: Zoomed-in image Consent was obtained from the volunteers for the use of the images.

For the anterior view, each retractor is held by the dental assistant [24]. The retractor is pulled laterally (Figure 19). The dental midline should be in the middle of the frame. If there is a dental midline discrepancy, the frenum or philtrum can serve as a guide. The teeth should be in maximal intercuspation. The buccal segment should be equally displayed bilaterally. The camera should be positioned perpendicular to the teeth. Any tilting of the camera might cause inaccuracies in the amount of overbite. If there is displacement due to premature contact, the teeth that are in contact should be recorded.

Buccal Photos

**Figure 20:**
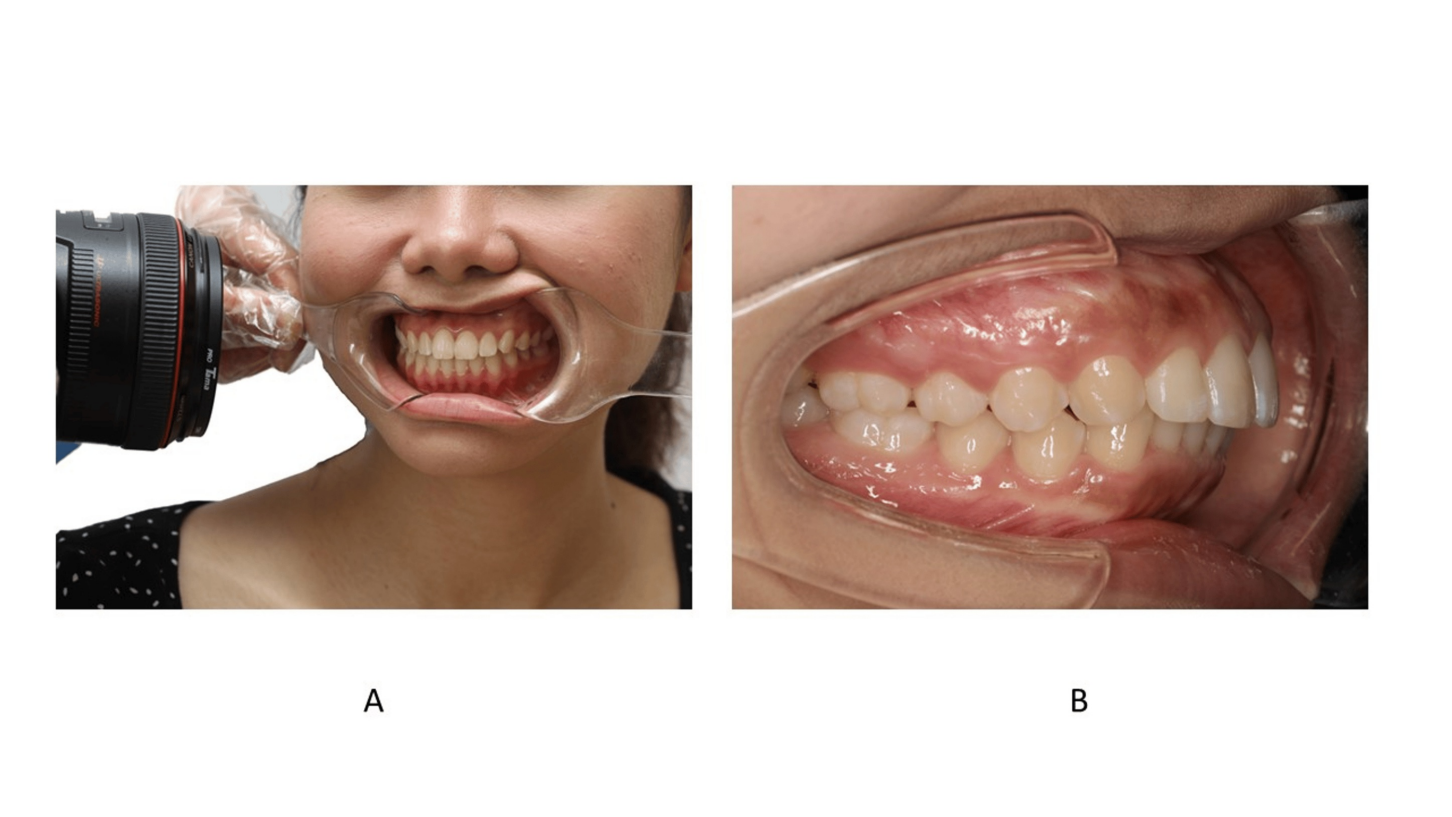
The position of the retractor during the buccal view. A: Front view; B: Zoomed-in image Consent was obtained from the volunteers for the use of the images.

For buccal shots, one retractor is held by the dental assistant and the other by the clinician (Figure 20). The patient is instructed to turn their head in the direction opposite the planned buccal shot. The other side of the retractor is held in place passively. During the retraction, the clinician should instruct the patient to resist the pulling force. The camera should be perpendicular to the buccal teeth [28], and the focus area should be at the canine or premolar region. The buccal photo should include the first molar. The authors do not prefer to use a mirror for the buccal shot because it prevents the clinician from taking a perpendicular photo.

Occlusal Photo

**Figure 21:**
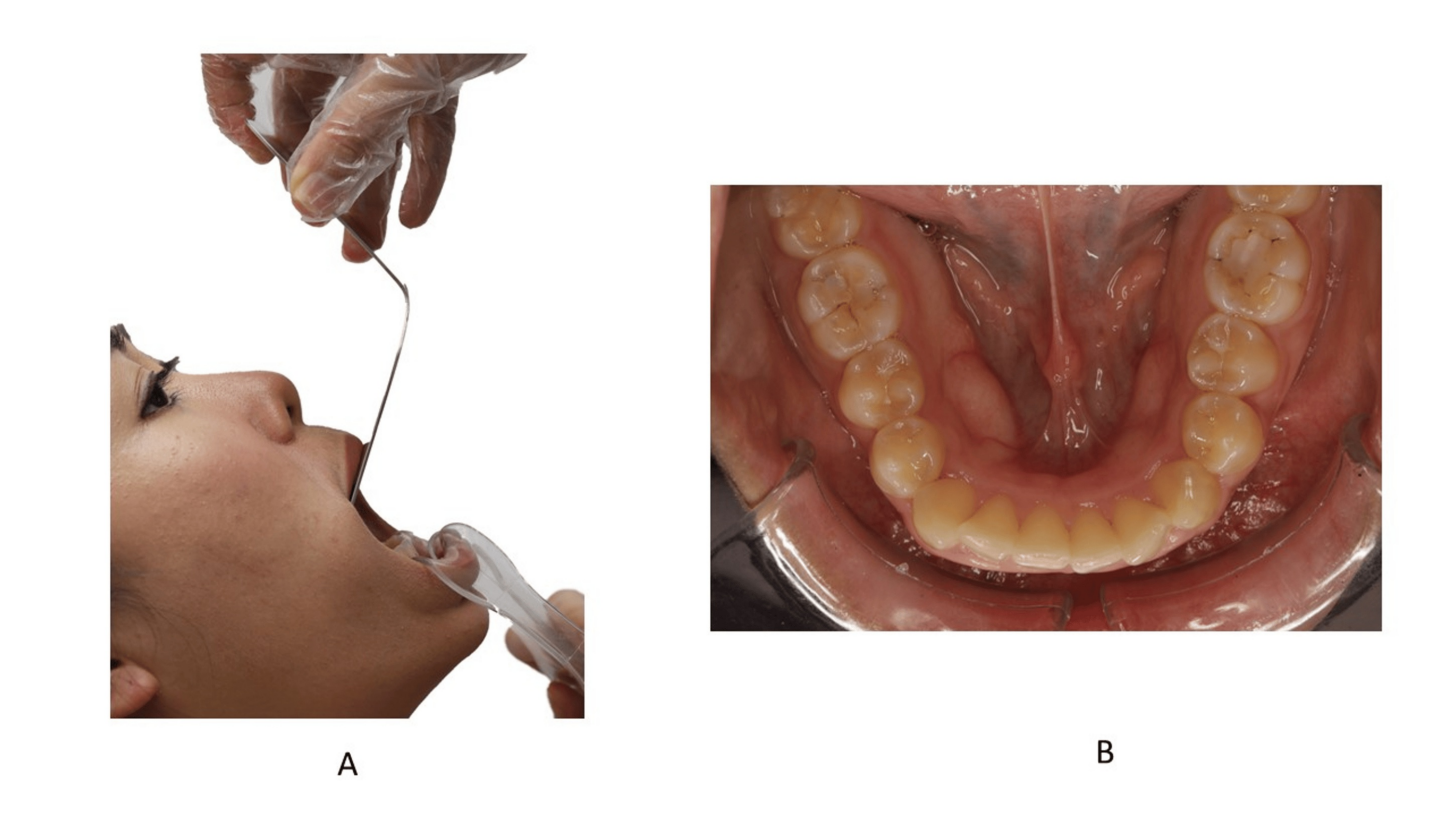
The position of the retractor during the occlusal view A: Front view; B: Zoomed-in image Consent was obtained from the volunteers for the use of the images.

For an occlusal view, the dental assistant holds the retractor (Figure 21) while the clinician holds the mirror [5]. For the lower teeth, the tongue should be positioned behind the mirror. Unlike the buccal and anterior photo, the retractor is pulled downward and outward, and the patient is advised to raise their chin to allow maximum opening and a better view of the occlusal surface. The camera should be positioned as perpendicular as possible in relation to the occlusal plane. The clinician should ensure the lower anterior lingual surface is seen in the photo. For the upper occlusal, the retractor is turned upward and outward. The mid-palatal raphe should be at the center of the frame. Because the tongue tends to push the mirror upward, the operator must resist the upward force by the tongue.

Discussion

**Figure 22:**
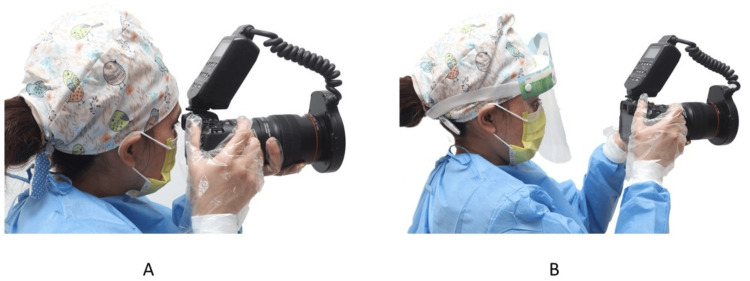
The position of the camera when using (A) the viewfinder and (B) back LCD monitor. Consent was obtained from the volunteers for the use of the images.

Due to the COVID-19 pandemic and the ease of spreading the virus to orthodontists, health authorities have recommended extra personal protective equipment (PPE), including a face shield [29]. When wearing a face shield, the author recommends using the LCD monitor on the back of the camera instead of the viewfinder (Figure 22). Looking through the viewfinder is difficult while wearing the face shield and having your hand near your face is best avoided. If the orthodontist has a weather-sealed camera and lenses, regularly cleaning the camera and the camera neck strap is advised.

The authors would like to emphasize that camera equipment, like any other dental equipment, may fail. Hence, regular maintenance of the system is mandatory. Due to the risk of equipment failure, the author prefers to use a modular set of camera equipment rather than a single system such as the EyeSpecial digital camera [30]. In the event of a failure of the camera, lens, or flash, the author can replace only the faulty item rather than the entire system.

Taking photos with any intraoral or extraoral functional or removable appliance follows the same technique. However, the clinician should take photos both with the appliance in place and without it. Certain adjustments may be necessary; for instance, an occlusal photo may not be feasible when the patient is wearing a twin block appliance, so it may be advisable to remove one arch of the appliance to allow better access for the intraoral mirror when taking the photo.

There is no denying that taking quality orthodontic clinical photos takes some time. In a busy practice, taking photos might become a chore for an orthodontist. One solution is to delegate the task to a dental assistant. A study by Sandler et al. [25] demonstrated that dental assistants are capable of taking quality orthodontic clinical photos. Training a dental assistant to take high-quality photos will allow the orthodontist to allocate their time to other clinical procedures.

## Conclusions

Mastering the necessary technique for taking quality orthodontic photographs does not happen overnight. However, with practice and the proper technique, any orthodontist should be more than capable of producing high-quality orthodontic clinical photos.
